# Analysis of Residual Stress in Electrical Penetration Assembly Based on a Fiber Bragg Grating Sensor †

**DOI:** 10.3390/s19010018

**Published:** 2018-12-21

**Authors:** Zhichun Fan, Xingzhong Diao, Yong Zhang, Malin Liu, Feng Chen, Zhiyong Huang, He Yan

**Affiliations:** 1 Institute of Nuclear and New Energy Technology, Key Laboratory of Advanced Reactor Engineering and Safety of Ministry of Education, Collaborative Innovation Center for Advanced Nuclear Energy Technology, Tsinghua University, Beijing 100084, China; fzc16@mails.tsinghua.edu.cn (Z.F.); diaoxz@tsinghua.edu.cn (X.D.); liumalin@mail.tsinghua.edu.cn (M.L.); fengchen@mail.tsinghua.edu.cn (F.C.); huangzy@tsinghua.edu.cn (Z.H.); 2 Institute of Nuclear and New Energy Technology, Beijing Key Laboratory of Fine Ceramics, Tsinghua University, Beijing 100084, China; yzhang@tsinghua.edu.cn

**Keywords:** sealing process analysis, residual stress, embeddable technology, fiber Bragg grating, electrical penetration assembly

## Abstract

An important factor for maintaining hermeticity of a metal-to-glass sealed electrical penetration assembly (EPA) is the residual stress in the sealing glass, which is generated during the EPA sealing process. A novel method to investigate and optimize the sealing process of EPAs, based on a fiber Bragg grating (FBG) sensor, is proposed in this research. An FBG was well bonded with sealing glass to measure the parameters of the glass during the sealing process. The temperature change during the heating process was able to be measured by Bragg wavelength shift. After the sealing glass solidified and dropped to room temperature, the residual stress was determined and the effect of temperature was minimized because the temperature before and after the sealing process was the same as room temperature. The curing temperature of the sealing glass was evaluated to specifically investigate the solidification process of the EPA. This study provides a basis for online stress and temperature monitoring of EPAs under external loads in nuclear power plants.

## 1. Introduction

**Figure 1:**
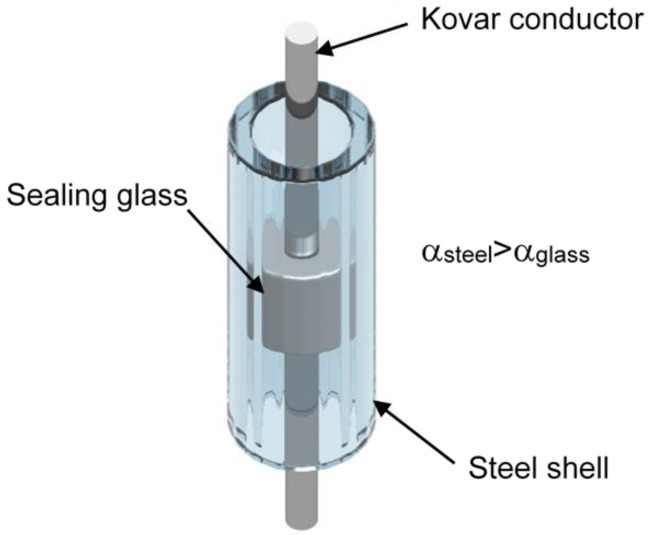
Diagram of an electrical penetration assembly (EPA) model in nuclear power plants.

Metal-to-glass seals have good hermeticity and can endure harsh environments, and they have been widely used in solar receivers [1,2], solid oxide fuel cells [3], and biomedical applications [4]. Such seals can be divided into two types—match sealing and mismatch sealing—according to the coefficient of thermal expansion (CTE) of metal and glass [1]. Different CTEs create residual stress in sealing glass to achieve good hermeticity. Electrical penetration assembly (EPA), which provides a pressure barrier for the containment structure in nuclear power plants, is a mismatch metal-to-glass sealing structure, as shown in Figure 1. If the residual stress is too small, the sealing glass and steel shell do not fully bond and hermeticity fails. Therefore, it is necessary to measure the residual stress in sealing glass and to optimize the sealing process of the EPA.

Due to the limited size of sealing glass in an EPA (2 mm thickness), the traditional strain gauge is not suitable, and many other measurement tools have limitations under this circumstance. Optical fiber sensing techniques have been applied to measure different parameters [5,6,7,8,9,10] (strain, temperature, deformation, humidity, etc.) and to simultaneously monitor multiple parameters [11,12,13,14] in many fields, such as aerospace engineering [15,16] and the power industry [17,18,19]. These techniques have shown better performance in comparison with conventional sensors. Optical fiber sensors have been chosen to monitor axial stress in sealing glass for two reasons. One is that fiber has a small diameter (about 0.1 mm), and hence it would not have an adverse effect on the compact structure. The other reason is that both fiber sensors and sealing glass have similar chemical contents, which allows them to easily bond together to precisely monitor sealing glass without affecting insulation or hermeticity. When compared with other optical fiber sensors, fiber Bragg grating (FBG) sensors have unique advantages, such as being easy to embed in the tested object, mature inscribing techniques, and are more suitable for strain measurement at high temperatures. 

Glass-to-metal sealing EPA research has been presented by Li et al. [20]; however, the EPA model in their research was a simplified model that only contained a steel shell and sealing glass, and the heating process was achieved by a high-frequency induction heater, which is not suitable for a complete EPA model with a Kovar conductor. The sealing process and related numerical simulation of a complete EPA model was carried out by Dai et al. [21,22,23], but the monitoring of residual stress was not able to be achieved while using a feasible method. 

In the present research, the sealing process of a complete EPA model was explored through pressure tests and scanning electron microscopy (SEM). An FBG sensor was embedded in sealing glass, so that they could bond together as the glass was heated to melting point and then allowed to solidify. The temperature change during the sealing process and the residual stress after the sealing process was measured using the Bragg wavelength shift, and the cure temperature of the sealing glass was obtained. Based on the preliminary work [24], a finite element model was built to simulate the compaction process and obtain the global stress distribution, which provided a reference to the experimental results. 

## 2. Experimental Setup

### 2.1. Investigations into the EPA Sealing Process

**Figure 2:**
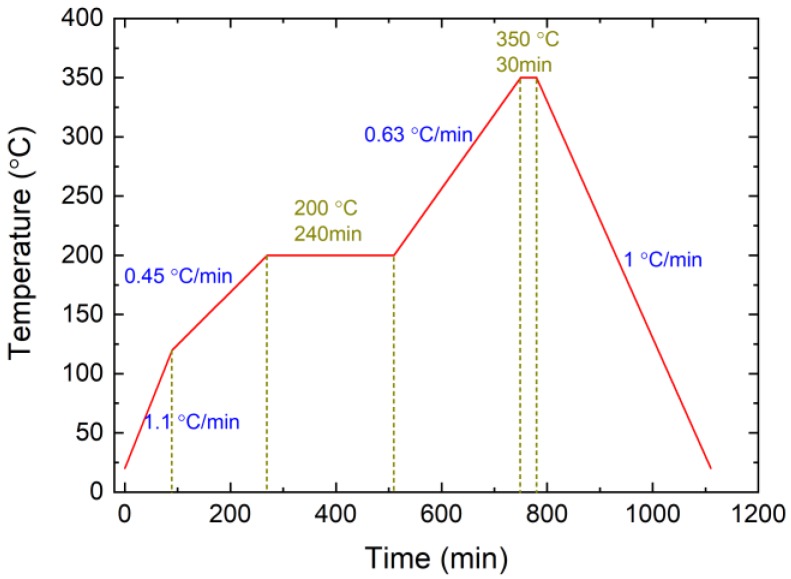
Sintering process of low-melting-point glass.

An EPA model was constructed from sealing glass, a steel shell, and a Kovar conductor, the same as was used in a real EPA in a nuclear power plant. The outer and inner diameters of the steel shell were 5 mm and 3.5 mm, and those of the sealing glass were 3.5 mm and 2.5 mm, respectively. The diameter of the Kovar conductor was 2.5 mm. The material of the steel shell was 316 stainless steel. The Kovar conductor was constructed from Kovar alloy. The sealing material was a low-melting-point glass. First, the granulated glass powder was formed into a cylinder with a central path for the Kovar conductor and axial paths for the penetration of the FBG. Subsequently, the glass cylinders were sintered after the heating process shown in Figure 2. 

**Figure 3:**
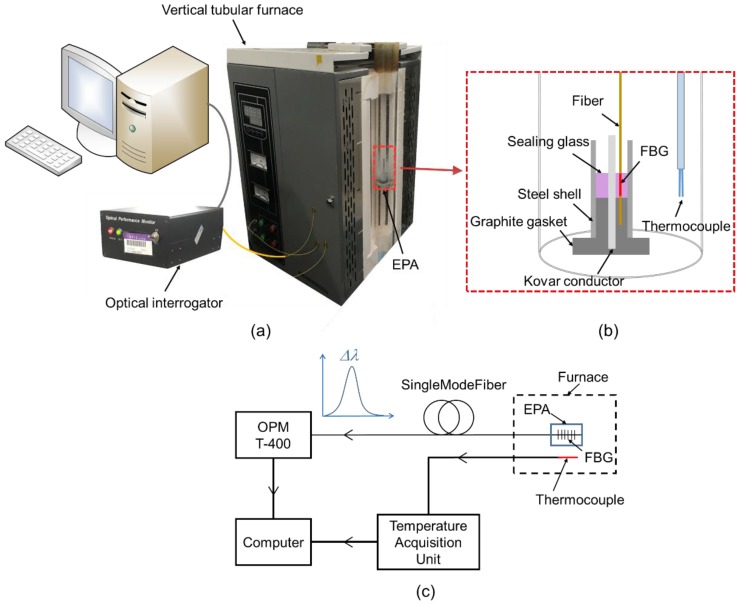
(**a**) Experimental setup for the sealing process analysis of the EPA. (**b**) Schematic of the assembled EPA model. (**c**) Diagram of the experimental setup.

**Figure 4:**
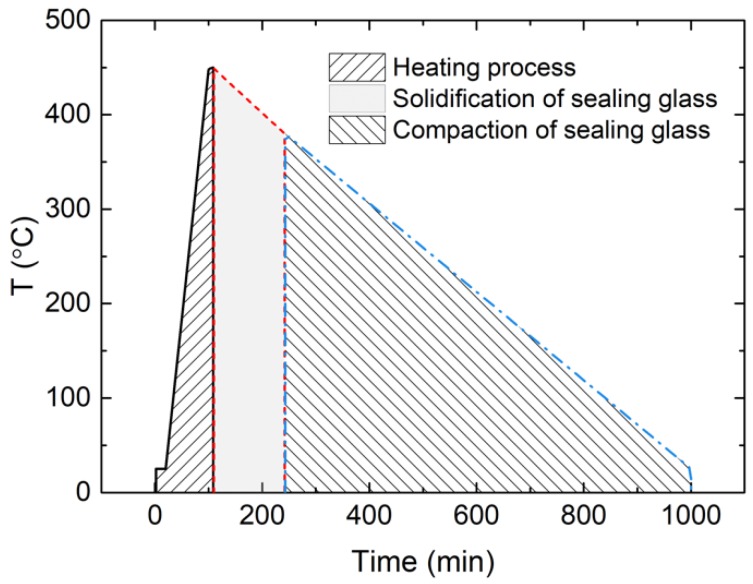
Three stages of the EPA sealing process.

The detailed experimental setup for investigation of the sealing process was as follows. The EPA was assembled, as shown in Figure 3a,b. A graphite gasket was used to maintain the location of the sealing glass and Kovar conductor in the middle of the steel shell. The assembled model was set into a vertical tubular furnace to conduct the sealing process shown in Figure 4.

To explore the appropriate sealing process and obtain EPA samples with qualified hermeticity, the EPA sealing processes were carried out at different heating temperatures (i.e., the maximum temperature of the sealing process), varying in a range of 420–500 °C. The complete EPA samples obtained at different temperatures were examined using a pressure test to ensure that the samples could sustain 8 MPa, which is the operational pressure in a nuclear reactor. Scanning electron microscopy (SEM) tests were carried out to observe the microstructure of different EPA samples to provide a detailed understanding of the sealing process.

### 2.2. Temperature Measurement During the Sealing Process

The maximum heating temperature is important for the hermeticity of EPAs. Therefore, an FBG sensor was embedded in the sealing glass to measure the temperature during the heating process. The FBG used in this research was an ultraviolet (UV)-laser-inscribed FBG on single mode fiber without a fiber coating, with a central wavelength of about 1549.673 nm. The grating length in the sealing glass was 5 mm to match the length of the glass cylinder. The FBG was connected to an optical interrogator to translate optical signals to Bragg wavelength data shown on a computer, as illustrated in Figure 3a. The optical interrogator OPM-T400 was used to characterize the FBG sensor, provided by Gaussian Optics (Wuhan, China). A standard K-type thermocouple was set near the EPA model and was used to monitor temperature as calibration of the results of the FBG.

Temperature could be measured during the heating process because the Bragg wavelength shift was only affected by temperature before the solidification process. The relationship between the Bragg wavelength shift Δ*λ_B_* and temperature change Δ*T* is shown in Equation (1) [25]:

(1) $$\frac{\Delta \lambda_{B}}{\lambda_{B}}\;=\;(\zeta +\alpha )\times \Delta T$$

where *ζ* is the thermo-optic coefficient, *α* is the coefficient of thermal expansion, and *λ_B_* is the initial Bragg wavelength of the FBG.

### 2.3. Measurement of the Residual Stress of the Sealing Glass

During the compaction process, the sealing glass was solidified completely and it began to generate residual stress due to the compaction of the steel shell. After the sealing process, the residual stress in the sealing glass could be demodulated by a single FBG. Because the temperatures before and after the sealing process were the same as room temperature, the effect of temperature on the Bragg wavelength could be minimized. Strain *ε* in the sealing glass can be calculated by Equation (2) [25]:

(2) $$\frac{\Delta \lambda_{B}}{\lambda_{B}}=(1-P_{e})\times \varepsilon$$

where *P_e_* is the strain-optic coefficient. Combined with Hook’s law, the stress *σ* could be obtained by multiplying *ε* and Young’s modulus.

### 2.4. Measurement of the Cure Temperature of the Sealing Glass

The sealing process of the glass in the EPA was divided into three stages: heating, solidification and compaction, as illustrated in Figure 4. Through measurement of temperature and residual stress, heating, and compaction during the sealing process could be studied. 

The solidification of the sealing glass is an important process during which the sealing glass maintains its molten state before dropping to cure temperature. Thus, the residual stress in the sealing glass would disappear if the EPA was reheated to cure temperature, and the hermeticity would be destroyed. The cure temperature could be used in finite element analysis instead of the maximum heating temperature to improve the accuracy of the numerical simulation. Hence, the cure temperature should be measured.

## 3. Numerical Simulation

### 3.1. Finite Element Model

The finite element model of the metal-to-glass sealed EPA was the same as the real model that is shown in Figure 1. Axial and radial paths were chosen as stress calculation paths. The mechanical properties of the materials are shown in Table 1.

### 3.2. Boundary Conditions and Mesh

The bottom surface of the steel shell was fixed as the displacement boundary condition. Contact between the steel shell, sealing glass, and Kovar conductor was bounded and non-slippery. A thermal load was added under the static structural module to simulate the compaction process of the EPA. Temperature was varied from cure temperature to room temperature. The global distribution of residual stress was a supplement and reference to the experimental results.

## 4. Results and Discussion

### 4.1. Results of Investigation into the EPA Sealing Process

**Figure 5:**
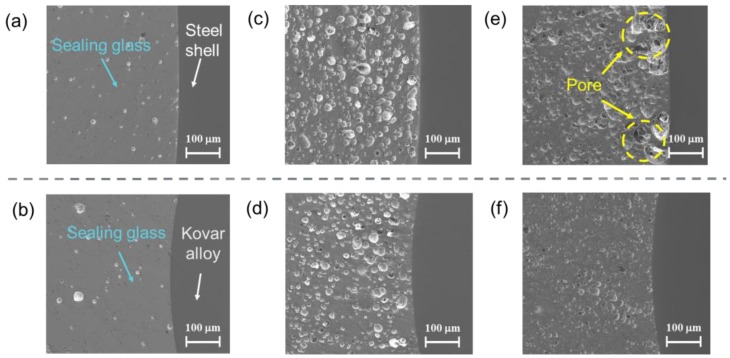
Microstructure of (**a**) sealing glass and steel shell with 450 °C EPA, (**b**) sealing glass and Kovar conductor with 450 °C EPA, (**c**) sealing glass and steel shell of 470 °C EPA, (**d**) sealing glass and Kovar conductor of 470 °C EPA; (**e**) microstructure of sealing glass and steel shell of 490 °C EPA; and, (**f**) microstructure of sealing glass and Kovar conductor of 490 °C EPA.

**Figure 6:**
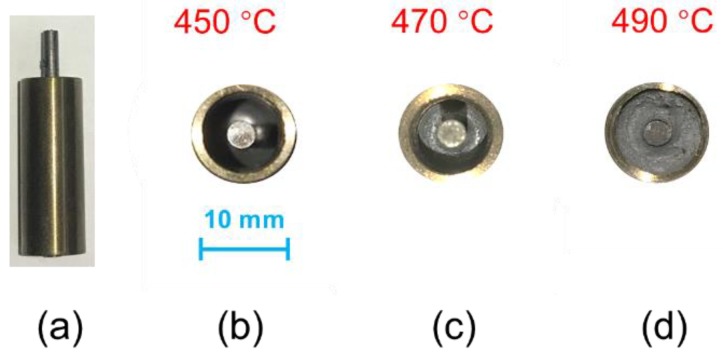
(**a**) Photograph of EPA sample after sealing process, (**b**) smooth and glossy surface of sealing glass, (**c**) surface of sealing glass with small bubbles, and (**d**) uneven surface of sealing glass.

A total of 27 samples, with heating temperature varying from 420 to 500 °C, were produced. All of the samples were examined by a pressure test, and test results are shown in Table 2. The microstructure of the cross-section of the sealing glass and the macrostructure of the EPA samples are shown in Figure 5 and Figure 6, respectively. 

The sealing glass bonded well with other parts of the 450 °C EPA sample, and it showed a flat surface with few bubbles. Many small bubbles were observed in the cross-section of the 470 °C sample without affecting the hermeticity of the EPA. The sealing structure of the 490 °C sample was porous, especially at the junction of the sealing glass and the steel shell, leading to the destruction of hermeticity. The reason for this might be that the compressive stress between the sealing glass and the steel shell was less than that at the junction of the sealing glass and the Kovar conductor, which could be analyzed numerically using the finite element method. If the temperature was below 450 °C, then the heat was insufficient to melt the sealing glass and to generate the residual stress required to achieve hermeticity. To unify the following experiments, 450 °C was considered as the standard temperature to achieve good hermeticity.

### 4.2. Results of Temperature Measurement

**Figure 7:**
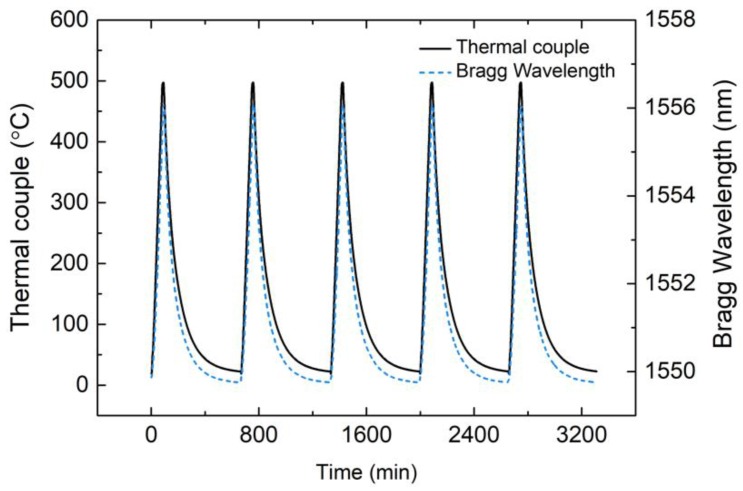
Temperature measurement results of the fiber Bragg grating (FBG) and thermocouple from the five-cycle heating test.

**Figure 8:**
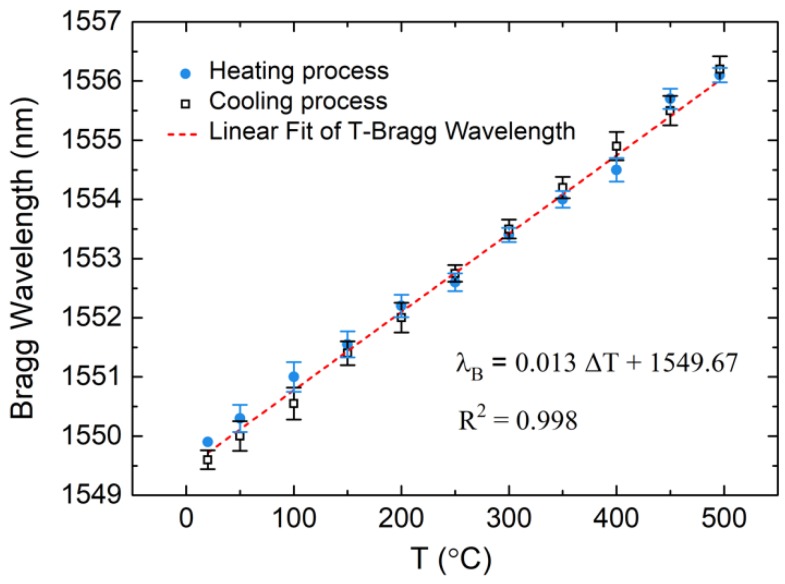
Relationship between temperature and Bragg wavelength obtained from test data.

Since the temperature of the heating process had a significant effect on the hermeticity of the EPA samples, the maximum heating temperature should be controlled at 450 °C to lay the foundation for residual stress measurement. To examine the feasibility of the FBG sensor for measuring temperature, a five-cycle heating test was carried out. The results of the Bragg wavelength curve and corresponding temperature are shown in Figure 7. Wavelength shift was consistent with temperature which was measured by thermocouple. The FBG sensor was better able to directly measure the temperature of the EPA compared with the thermocouple, because it was embedded in the sealing glass. The temperature and corresponding Bragg wavelength data from the five-cycle test are shown in Figure 8 to define the relationship.

The results showed little deviation and had good repeatability and accuracy. The thermal sensitivity index of the FBG was about 0.0132 nm/°C, which could also be calculated using Equation (3). The thermo-optic coefficient *ζ* and the coefficient of thermal expansion *α* were 6.7 × 10^−6^ and 5.5 × 10^−7^ [25], respectively, and the initial Bragg wavelength was about 1550 nm, so the theoretical thermal index was about 0.0112 nm/°C. The experimental and theoretical results showed minimal differences in the thermal sensitivity index; the experimental value also had good repeatability during the five-cycle heating tests. Hence, the FBG is a feasible method to achieve accurate temperature measurements in the EPA heating process.

### 4.3. Results of Residual Stress Measurement

**Figure 9:**
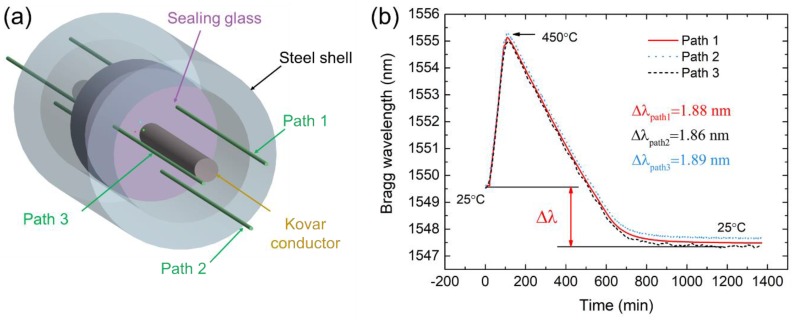
(**a**) Setup of three FBGs in the same sealing glass; (**b**) Bragg wavelength curve during the sealing process with wavelength shift standing for residual stress in the sealing glass.

To eliminate measurement deviation induced by positions of the FBG sensor in the sealing glass, three identical FBGs with 5-mm gratings were uniformly embedded in the sealing glass, as shown in Figure 9a, with the measuring results shown in Figure 9b. When the FBG experienced a temperature change and then returned to the original temperature, the Bragg wavelength would undergo a tiny change due to wavelength drift, Δ*λ_drift_*. The Δ*λ_drift_* of the FBGs used in this research was 0.14 nm, as determined from the temperature measurement test. The residual stress could be determined by subtracting Δ*λ_drift_* from the Bragg wavelength shift. Shift between the initial and final wavelength was defined as Δ*λ_residual_*. Equation (2) could be converted to Equation (3), as *P_e_* was 0.22 and the unit of *ε* was με. Results measured at different locations were almost the same, at around 1.88 nm, and the corresponding residual stress was about 80 MPa.

(3) $$\varepsilon =\frac{\Delta \lambda_{\mathrm{residual}}-\Delta \lambda_{drift}}{0.001209}$$

### 4.4. Results of Cure Temperature Estimation

The cure temperature of this low-melting-point glass was a certain value, T_c_, below 400 °C, which meant that, if the sealing glass was reheated to T_c_, the residual stress would disappear and the Bragg wavelength shift should be 1.88 nm, as measured by the 5 mm FBG sensor. Similarly, if the sealing glass was reheated to 400 °C, then the residual stress would transition from compressive stress to tensile stress, and the wavelength shift would be larger than 1.88 nm. Based on that, the cure temperature could be obtained by the reheating test.

**Figure 10:**
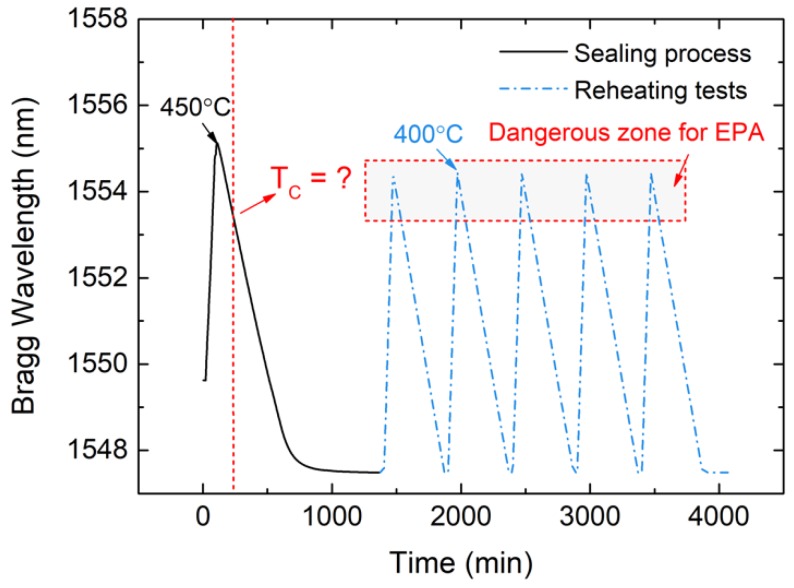
Relationship between temperature and Bragg wavelength obtained from test data.

A five-cycle reheating test was carried out after the EPA sealing process, and the Bragg wavelength curve is shown in Figure 10. Wavelength changes were almost the same during the five-cycle test, which means that the sealing structure was not destroyed and the results are reliable. The mean value of the Bragg wavelength shift Δ*λ_total_* of the five reheating tests was about 6.85 nm. The Bragg wavelength shift Δ*λ_total-T_* caused by temperature rising from 25 °C to 400 °C was calculated based on the results of the temperature measurement test, and the value was about 4.95 nm. The Bragg wavelength shift that was caused by changes in stress Δ*λ_total_-_σ_* was obtained by subtracting Δ*λ_total_* from Δ*λ_total-T_*, which was about 1.9 nm. Given that stress changed linearly with rising temperature, the relationship between Bragg wavelength shift caused by stress and temperature can be summarized by Equation (4).

(4) $$\frac{\Delta \lambda_{T_{C}}}{\Delta \lambda_{total–T}}=\frac{\Delta \lambda_{residual}}{\Delta \lambda_{total–\sigma }}$$

**Figure 11:**
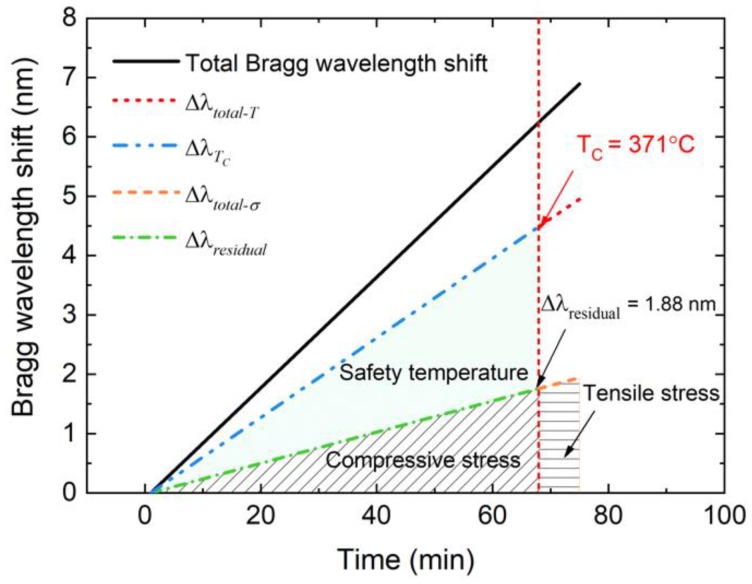
Calculation of the cure temperature from Bragg wavelength shift.

This calculation principle is illustrated in Figure 11. Δ*λ_Tc_* was 4.89 nm, which indicated the cure temperature T_c_, was about 371 °C. When the temperature of the sealing glass was higher than 371 °C, the residual stress in the sealing glass was released and the hermeticity was destroyed, because the tensile strength of glass is much smaller than the compressive strength. It would be dangerous for the EPA to work above 370 °C.

### 4.5. Results of Numerical Simulation

**Figure 12:**
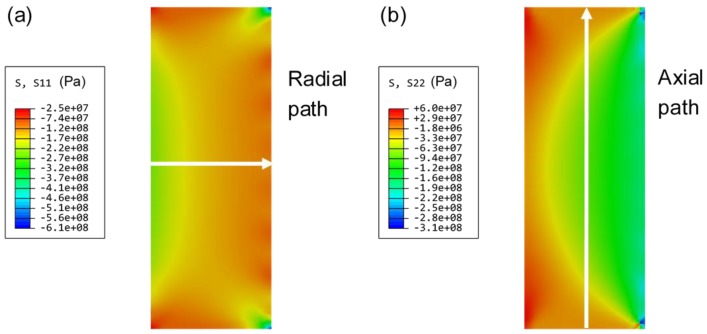
(**a**) Radial stress and (**b**) axial stress vector-graph of the sealing glass.

Numerical simulation was carried out to provide theoretical support for the stress measurement results in Section 4.3. Radial and axial stress vector-graphs of sealing glass were also compiled, as shown in Figure 12a,b, respectively. The radial stress was compressive and larger than the axial stress. The direction of axial stress near the inner surface of the sealing glass was tensile, and the stress inside the sealing glass and near the outer surface was compressive, which were affected by the expansion of the Kovar conductor and the compacting of the steel shell, respectively. The compressive stress was dominant in the sealing glass, leading to the hermeticity of the EPA. Stress along the radial and axial paths was extracted to perform a detailed analysis. The axial path was the central axis of the sealing glass where the FBG was exactly located in the experiment. The radial path was perpendicular to the inner wall of the steel shell and it passed through the center of the sealing glass.

**Figure 13:**
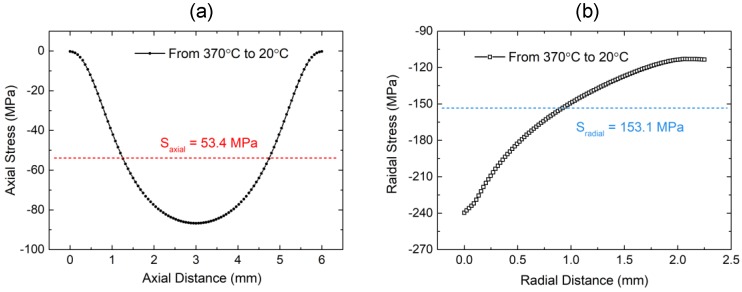
(**a**) Axial stress and (**b**) radial stress distribution in the sealing glass after the sealing process.

Residual stress calculated in axial and radial paths is shown in Figure 13a,b, respectively. Axial stress was symmetrical and maximized in the middle position. The mean value of axial stress was about 53.4 MPa, showing a deviation of about 30% as compared with the experimental result of 80 MPa measured by a 5-mm FBG sensor. Radial stress decreased linearly from the inner surface to the outer, and the average was about 153.1 MPa. This was consistent with the SEM result, which proved that the stress at the junction of the sealing glass and the steel shell was much smaller than that between the Kovar conductor and the sealing glass, leading to the porous structure at the junction of the glass and the steel shell and destruction of hermeticity.

## 5. Conclusions

A detailed investigation of the sealing process of an EPA was completed using a novel method based on an FBG sensor. During the sealing process, the heating temperature and cure temperature were measured by an FBG, which was essential for the control and optimization of the whole sealing process. Residual stress in the glass after the compaction process was also obtained by the proposed method. Based on temperature and residual stress measurement experiments, the FBG was found to be feasible for application in the manufacturing process of equipment for nuclear power plants. An attempt to measure the residual stress of a high-melting-point (900 °C) glass sealing EPA is involved in the future work.

## Figures and Tables

**Table 1:** Mechanical properties of different components of the EPA.

Property	316 Steel	Kovar Alloy	Glass
Density (g/cm^3^)	7.98	8.30	2.75
Young’ modulus (GPa)	183	157	56.5
Poisson ratio	0.3	0.3	0.25
Coefficient of thermal expansion (1/°C)	1.6 × 10^−5^	4.9 × 10^−6^	8.9 × 10^−6^

**Table 2:** Performance of EPA samples.

Heating Temperature (°C)	Surface Morphology of Sealing Glass	Hermeticity (8 MPa for 24 h)
420	Sealing glass had melted, but not fused with steel shell and Kovar conductor	Failed
430	Smooth and glossy	Failed
440	Smooth and glossy	Succeeded
450	Smooth and glossy	Succeeded
460	Small bubbles on the glass surface	Succeeded
470	Small bubbles on the glass surface	Succeeded
480	Small bubbles on the glass surface	Succeeded
490	Glass surface was uneven and not flat	Failed
500	Glass surface was uneven and not flat	Failed
